# Preclinical, non-genetic models of lung adenocarcinoma: a comparative survey

**DOI:** 10.18632/oncotarget.25668

**Published:** 2018-07-17

**Authors:** Florian Janker, Walter Weder, Jae-Hwi Jang, Wolfgang Jungraithmayr

**Affiliations:** ^1^ Department of Thoracic Surgery, University Hospital Zurich, Zurich, Switzerland; ^2^ Department of Thoracic Surgery, Brandenburg Medical School, Neuruppin, Germany

**Keywords:** lung cancer, mouse model, urethane, heterotopic, orthotopic

## Abstract

Lung cancer is the leading cause of cancer-related mortality worldwide. Animal models are key in analyzing cancer biology and therapy evaluation.

We here compared relevant non-genetic lung cancer models with regard to tumor induction period, incidence, morbidity and mortality rate and the immunological composition of primary tumors and the occurrence of tertiary lymphoid organs (TLO): (I) intraperitoneal Urethane injection (1g/kg), (II) Lewis lung carcinoma (LLC) cell line model (intravenous or subcutaneous), and (III) *ex vivo* three-dimensional (3D) primary cell culture model established from subcutaneously developed LLC-induced tumors.

The incidence of Urethane induced lung tumors was 100% in both, C57BL/6 and BALB/c strains without morbidity or mortality at twenty weeks after injection. The mean size of tumor nodules after Urethane injection was significantly larger in BALB/c mice vs. C57BL/6 (p<0.01). Three times of Urethane injection produced significantly more tumor nodules in both mouse strains compared to one injection (BALB/c: p<0.01; C57BL/6: p<0.05). TLOs were only found in the Urethane-induced model. Although the cell line models also showed 100% induction rate, morbidity was high due to skin ulceration on the inoculation site and the development of pleural effusions in the subcutaneous model and the intravenous model, respectively. Tendencies, but no significant differences (p>0.05) could be found in the count of CD4^+^, CD8^+^, F4/80^+^ and NKp46^+^ cells in a tumor nodule among investigated models.

All discussed models provided a high tumor incidence rate. TLOs were exclusively found in the Urethane-induced model. No significant difference could be found regarding immune cells across models.

## INTRODUCTION

Mouse tumor models have been proven to be crucial to understand cancer biology and to develop therapeutics against cancer [1]. There are, on the one hand, genetically engineered mouse models available when tumors develop upon induction of oncogenes or through the suppression of tumor suppressor genes [2, 3]. On the other hand, there are conventional lung cancer models using xeno- or allotransplantation of established cancer cell lines or carcinogens for the induction of cancer, either orthotopic in the original organ or heterotopic outside the organ [4]. The latter models are among the most frequently used models for the research of cancer mechanism and for preclinical drug development [1, 2].

One of those aforementioned mouse models is the chemical-induced cancer model. When using a susceptible mouse strain [5, 6], Urethane as a carcinogen induces lung cancer [7]. Urethane is known to activate the Kras proto-oncogene in early stages of murine lung tumor development and qualifies as such as an appropriate study model of Ras-driven lung cancer [8, 9]. Another lung cancer model employs the injection of lung cancer cell lines intravenously into a mouse to induce tumor growth either orthotopically or heterotopically. Both, human cancer cells and murine cancer cells can be employed in this model. Patient xenograft models using human cancer cells are usually applied to predict the drug response in patients [10]. Apart from these *in vivo* models, a three dimensional (3D) *ex vivo*/*in vitro* cell culture model is available as an emerging model technic with applications in cancer cell biology, drug discovery [11] and prediction of drug response in patients. This method was shown to be effective in different cancer entities, including lung cancer [12, 13]. This 3D culture model is able to mimic tumor biology, tumor microenvironment, cell-cell and cell-extracellular matrix interactions similar to *in vivo* tumors.

This study shall give a comparative analysis on the most frequently used non-genetic lung cancer models: the (I) Urethane-induced tumor model and the (II) cell line induced tumor models through intravenous or subcutaneous tumor cell injection and the (III) *ex vivo*/*in vitro* 3D primary cell culture model.

## RESULTS

### Urethane induced lung adenocarcinoma model

**Figure 1:**
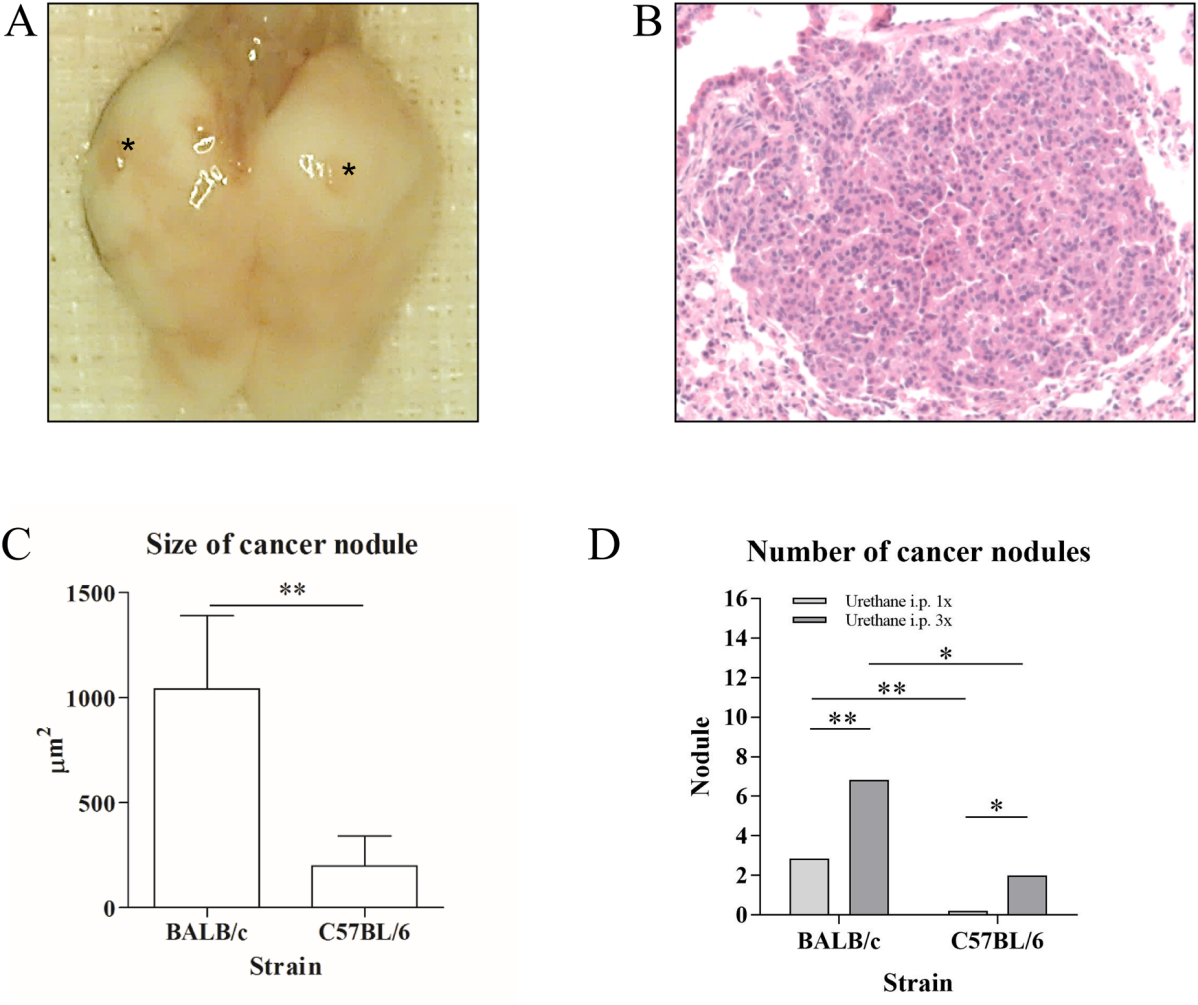
Representative macroscopic pictures **(A)** and H/E stains (picture was taken at x200 magnification) **(B)** of Urethane-induced tumor model. ^*^ indicates tumor nodule induced by intraperitoneal (i.p.) injection of Urethane. The nodules were found to be significantly larger in the BALB/c strain than in C57BL/6 (BALB/c: mean 1042μm^2^, C57BL/6: mean 199μm^2^; n=10 each group) **(C)**. Three injections of Urethane in one week developed significantly more nodules in BALB/c and C57BL/6 mice (BALB/c: mean 3 nodules in 1x injection of Urethane, mean 7 nodules in 3x injection; C57BL/6: mean 0 nodules in 1x injection of Urethane, mean 2 nodules in 3x injection; n=20 each group) **(D)**. ^*^p<0.05 and ^**^p<0.01.

**Figure 2:**
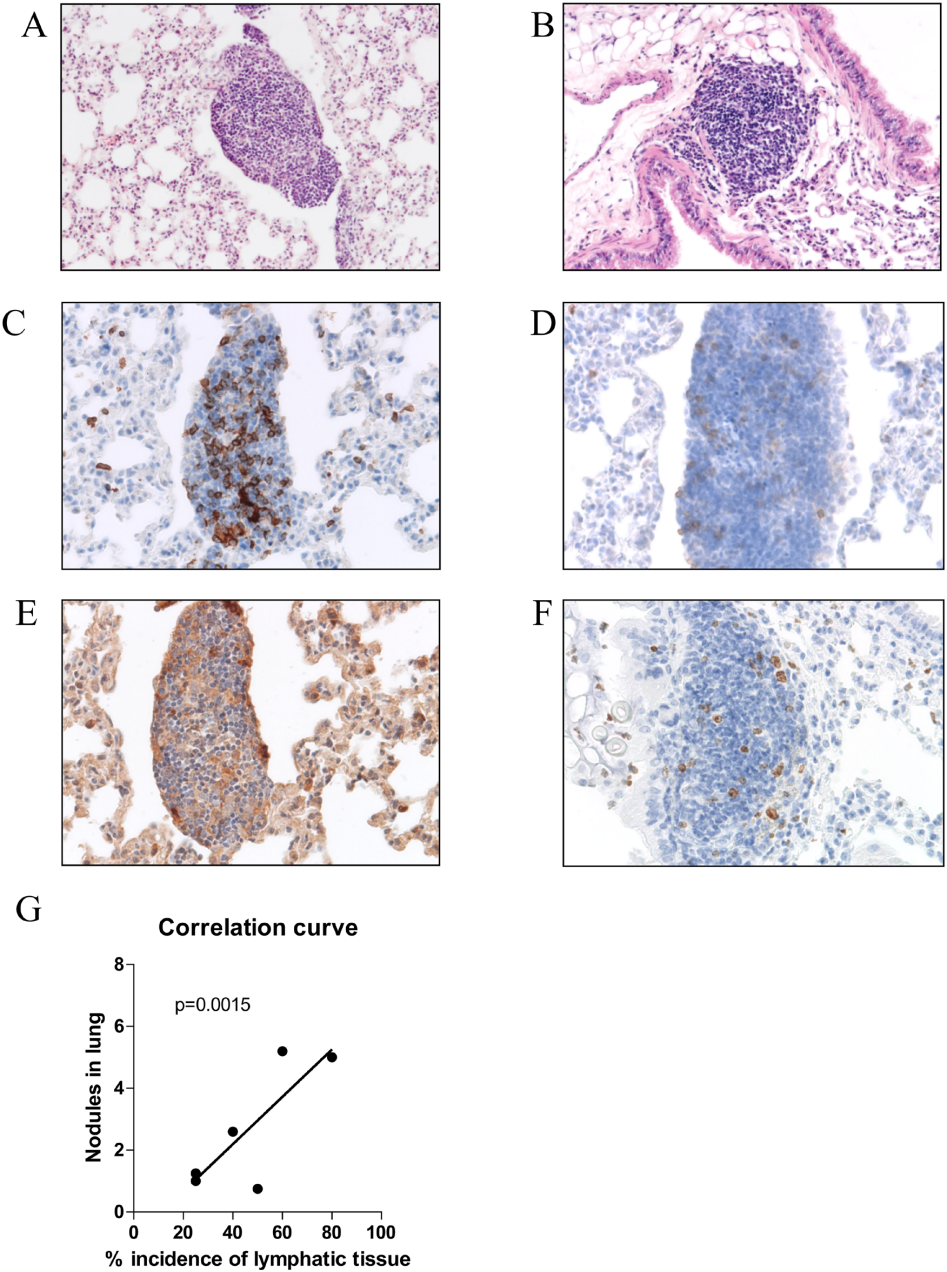
Features of Urethane induced lung cancer model Tertiary lymphoid organ was found in Urethane injected mouse lungs. It was heterogeneously located in alveolar **(A)** and/or perivascular regions **(B)**. Immunohistochemistry showed infiltrated immune cells expressing CD4 **(C)**, CD8 **(D)**, F4/80 **(E)**, and Ki-67 **(F)** (x200 magnification). The incidence of TLO significantly correlated (p=0.0015) with the number of tumor nodules in the lung **(G)**.

Twenty weeks after Urethane administration, we found heterogeneously developed tumor nodules within the lungs of mice (macroscopy Figure 1A, histology in Figure 1B). The tumor size and the count of tumor nodules were significantly higher in the BALB/c mouse strain than in the C57BL/6 mouse strain (Figure 1C and 1D). Three times of Urethane injection induced significantly more tumor nodules than one time injection in both mouse strains (Figure 1D) in spite of 0% mortality and morbidity with 100% tumor incidence rate. Interestingly, Tertiary lymphoid organs (TLOs) were exclusively found in the Urethane-induced tumor model compared to other models (microscopy in Figure 2A-2F) with a significant correlation of TLO incidence and the number of tumor nodules within the lung (Figure 2G). The location of TLO was in either alveolar (Figure 2A) or perivascular (Figure 2B) region with immune compositions of CD4^+^ T-cells (Figure 2C), CD8^+^ T-cells (Figure 2D), and macrophages (Figure 2E).

### Cell line induced lung cancer model via intravenous injection of LLC

**Figure 3:**
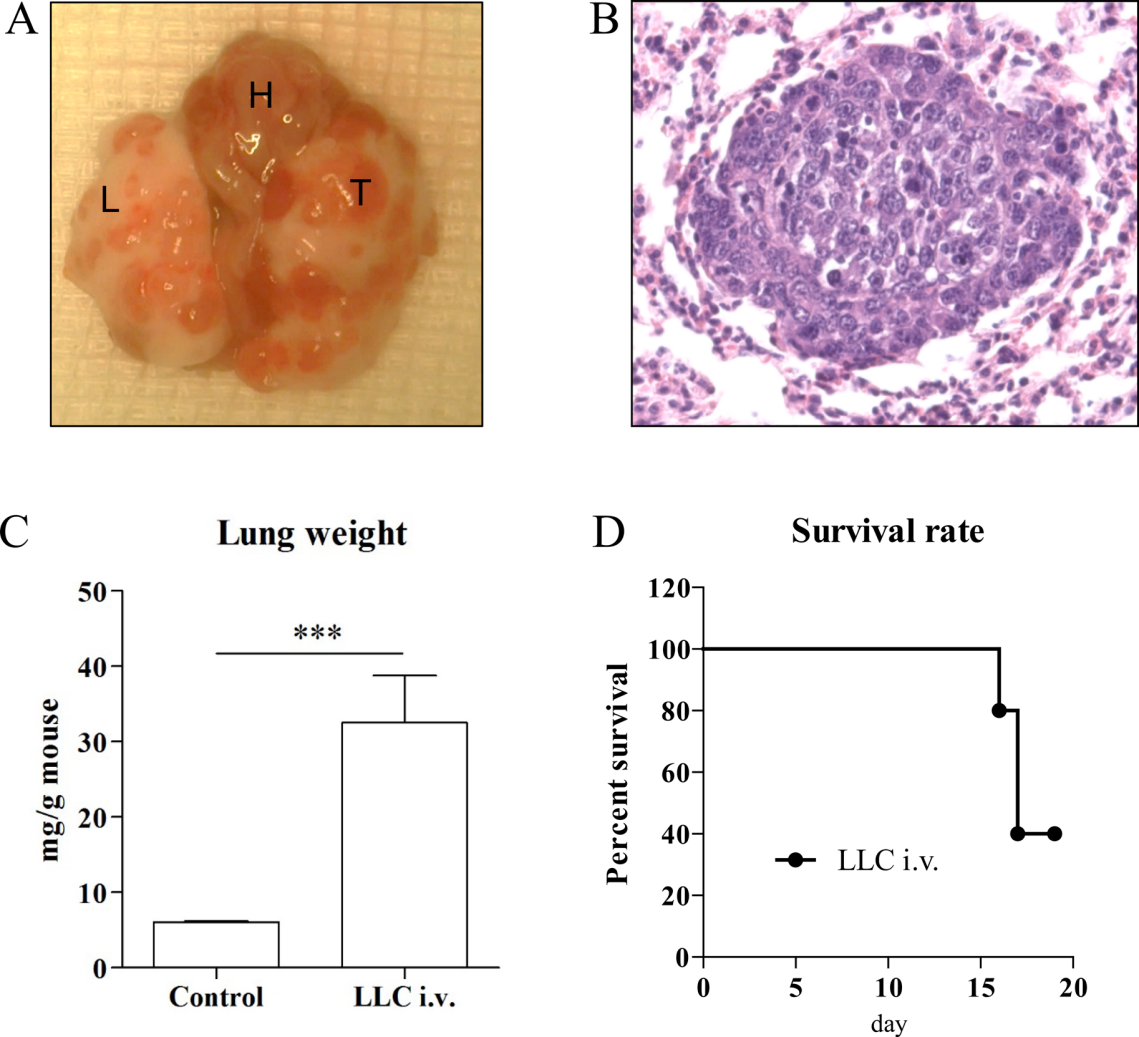
Representative macroscopic pictures **(A)** and H/E stains (x200 magnification) **(B)** of intravenous injection of tumor cell line model. T indicates tumor, L indicates lung, and H indicates heart. Intravenous injected LLC cell line developed diffuse lung cancer nodules two weeks after injection (n=10). Lungs (n=4) in tumor cell line-injected mice were significantly heavier two weeks after injection compared to control group (n=3) **(C)**. Pleural effusion-associated morbidity and mortality of LLC i.v. injected mice was observed in 60% of total (n=5) **(D)**. ^***^p<0.001.

Intravenously (i.v.) injected LLC cell line to syngeneic C57BL/6 mice developed orthotopic lung cancer in two weeks (macroscopy Figure 3A, histology Figure 3B). The weight of lungs with i.v. induced tumor was significantly heavier compared to the control group (Figure 3C). The tumor incidence by i.v. injection of LLC cell line reached 100%, in which nodules were diffusely distributed throughout the lung. Unlike other models, this model has a mortality rate of 60% with severe pleural effusion when extending the experiment to four weeks (Figure 3D).

### Cell line induced lung cancer model via subcutaneous injection of LLC

**Figure 4:**
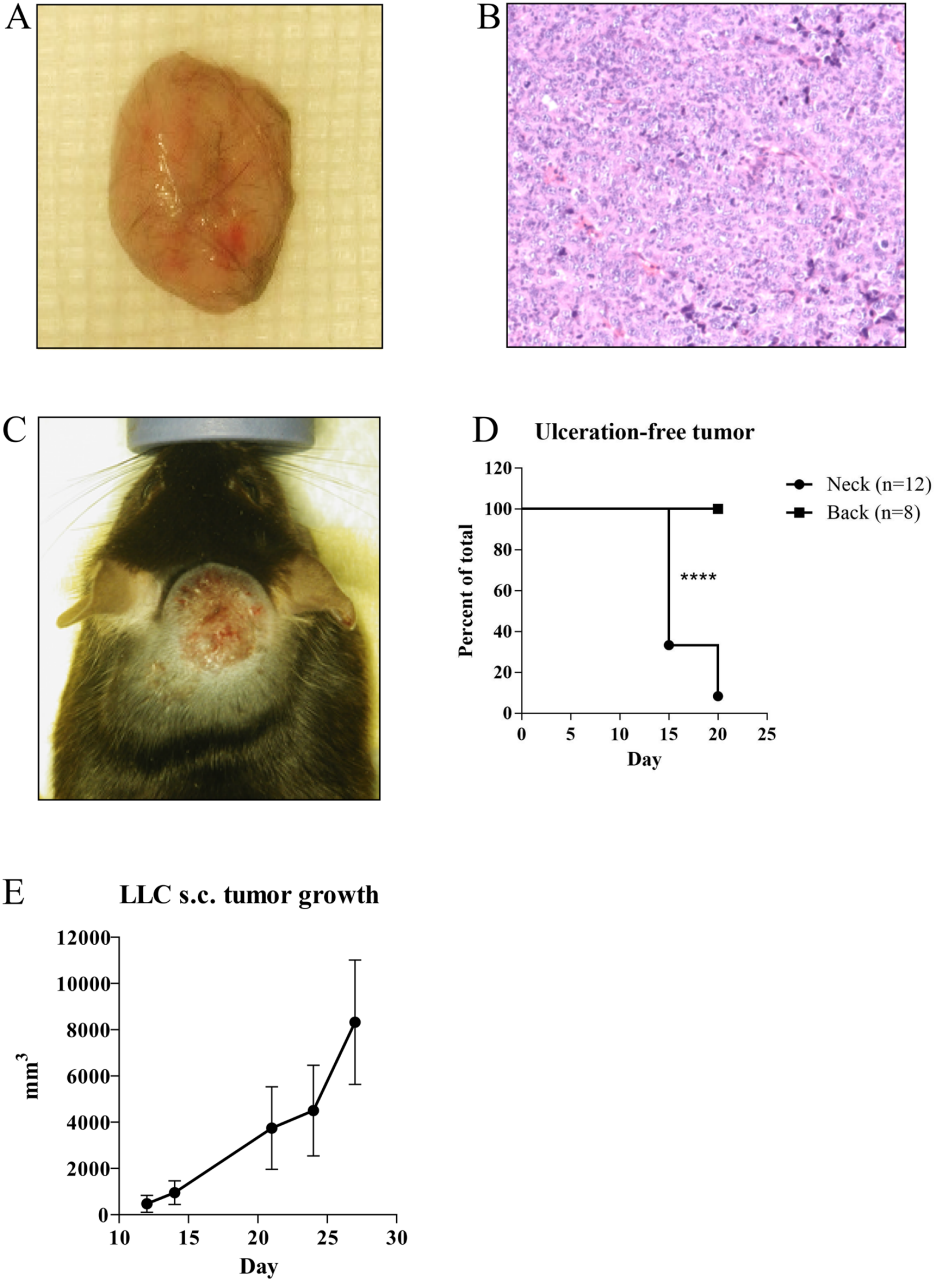
Representative macroscopic pictures **(A)** and H/E stains (x200 magnification) **(B)** of subcutaneous injection of tumor cell line model. Significant more skin ulcerations (n=12) **(C)** were found after subcutaneous tumor cell injection in neck compared to subcutaneous injections into back area (n=8) **(D)**. Exponential tumor growth developed by LLC s.c. injection was presented as mm^3^ volume measured by caliper (n=4) **(E)**. ^****^p<0.0001.

The tumor incidence rate with LLC s.c. injection reached 100% without mortality (macroscopy Figure 4A, histology Figure 4B). However, the morbidity of tumor ulceration was associated with the location of tumor cell injection. Tumors developed on the neck area caused a high rate of skin ulceration (macroscopy Figure 4C; 40% skin ulcerations after 15 days after injection, 80% ulcerations after twenty days after injection) (Figure 4D). Injections into the back area did not show a significant morbidity up to twenty days after inoculation (Figure 4D). Alternative assessment of s.c. tumor growth was performed by using caliper (Figure 4E) for four weeks after injection. Three dimensional s.c. tumor volume of each mouse is presented in Figure 4E.

### *Ex vivo* 3D tumor culture model

**Figure 5:**
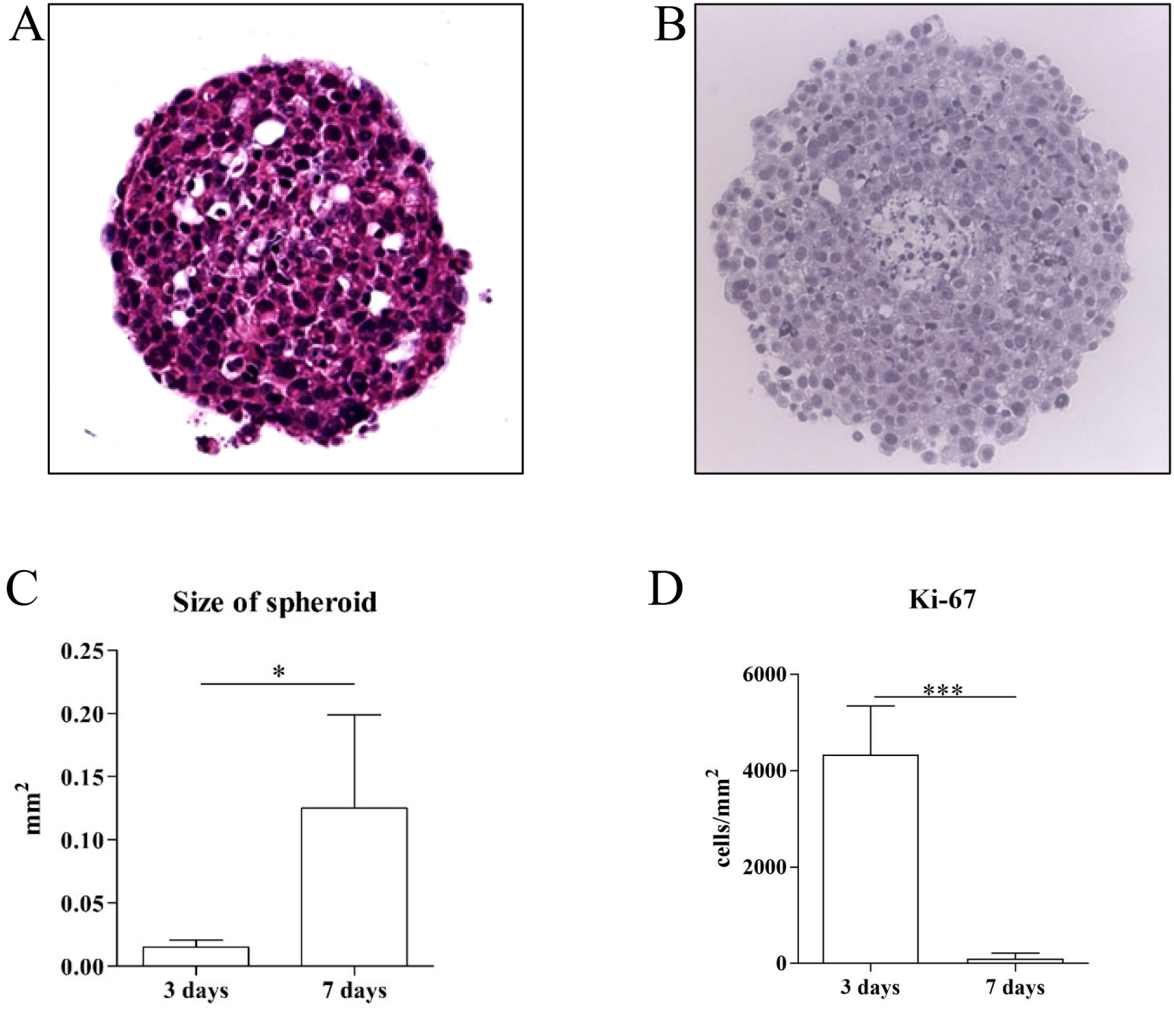
Representative pictures of H/E stains (x400 magnification) **(A)** of 3D primary tumor cell culture model after three and seven days of culturing **(B)**. Spheroids seven days after seeding were significantly larger in site compared to three days after seeding (n=4 each group) **(C)**. Spheroids contained significantly more proliferating cells (Ki-67^+^ cells) three days after seeding compared to seven days after seeding (n=4 each group) **(D)**. ^*^p<0.05 and ^***^p<0.001.

Single cell suspension of tumor cells obtained from the s.c. - induced tumor model formed spheroids three days after seeding in a 3D culture condition (Figure 5A). The size of the formed spheroids significantly increased seven days after seeding compared to three days after seeding (Figure 5B, and 5C) which was closely related with the degree of proliferation (Ki-67) (Figure 5D).

### Comparison among models

**Figure 6:**
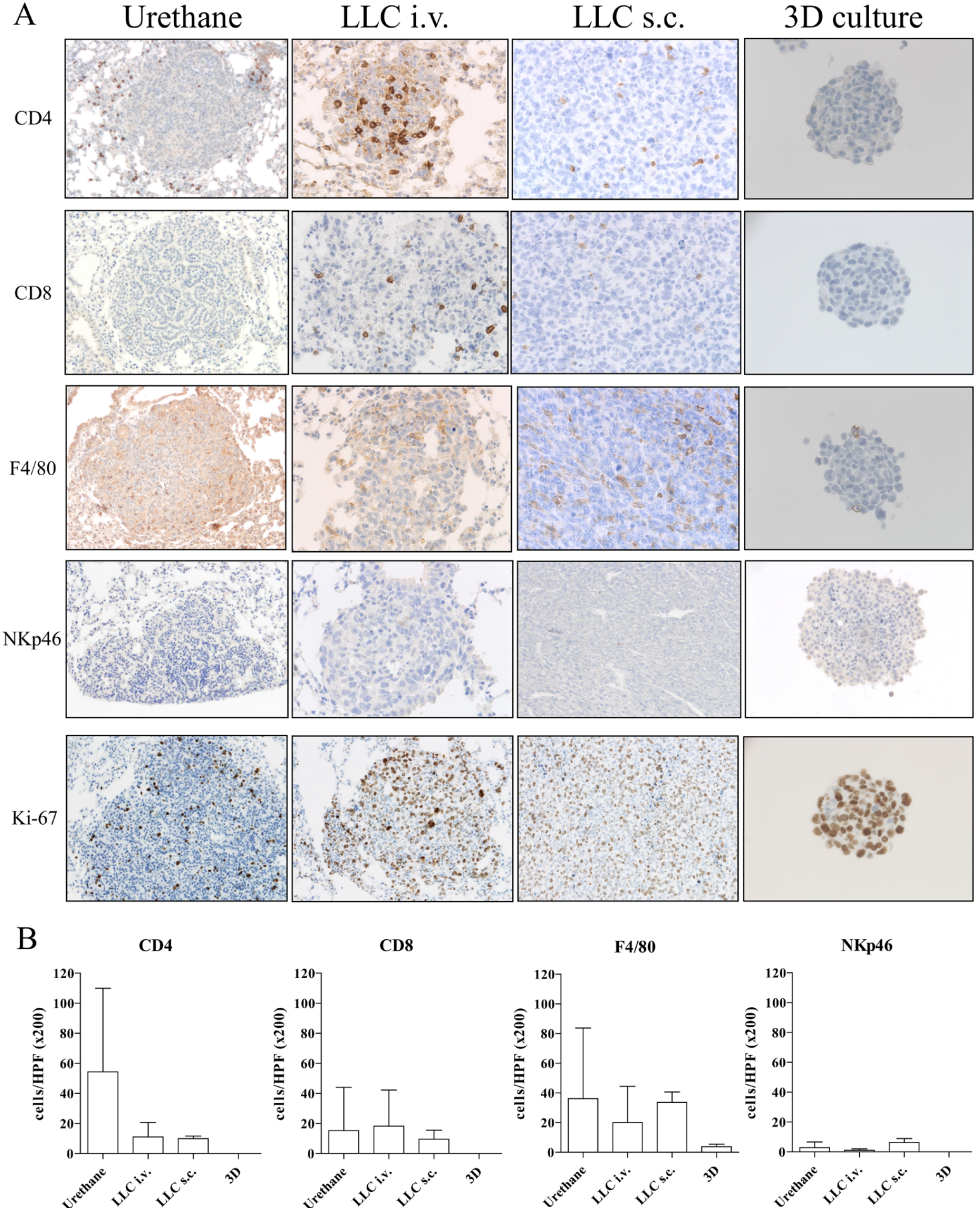
Immunohistological analysis of animal lung cancer models (x200 magnification for Urethane, LLC i.v. and LLC s.c. models and at x400 magnification for 3D primary cell culture model) **(A)** Urethane, LLC i.v., LLC s.c. induced lung tumor samples and 3D primary cell culture model spheroids were stained by antibodies of CD4, CD8, F4/80, NKp46, and Ki-67. **(B)** Comparison of microscopically counting of infiltrated CD4^+^, CD8^+^, F4/80^+^ and NKp46^+^ cells among models. Mean CD4^+^ cells per HPF at x200 magnification: 54 in Urethane model (n=6), 11 in LLC i.v. (n=11), 10 in LLC s.c. (n=7), 0 in 3D (n=4). Mean CD8^+^ cells per HPF at x200 magnification: 15 in Urethane model (n=7), 18 in LLC i.v. (n=9), 10 in LLC s.c. (n=8), 0 in 3D (n=4). Mean F4/80^+^ cells per HPF at x200 magnification: 36 in Urethane model (n=8), 20 in LLC i.v. (n=13), 34 in LLC s.c. (n=9), 4 in 3D (n=4). Mean NKp46^+^ cells per HPF at x200 magnification: 3 in Urethane model (n=7), 1 in LLC i.v. (n=13), 6 in LLC s.c. (n=4), 0 in 3D (n=4). p>0.05; HPF: high-power field.

**Figure 7:**
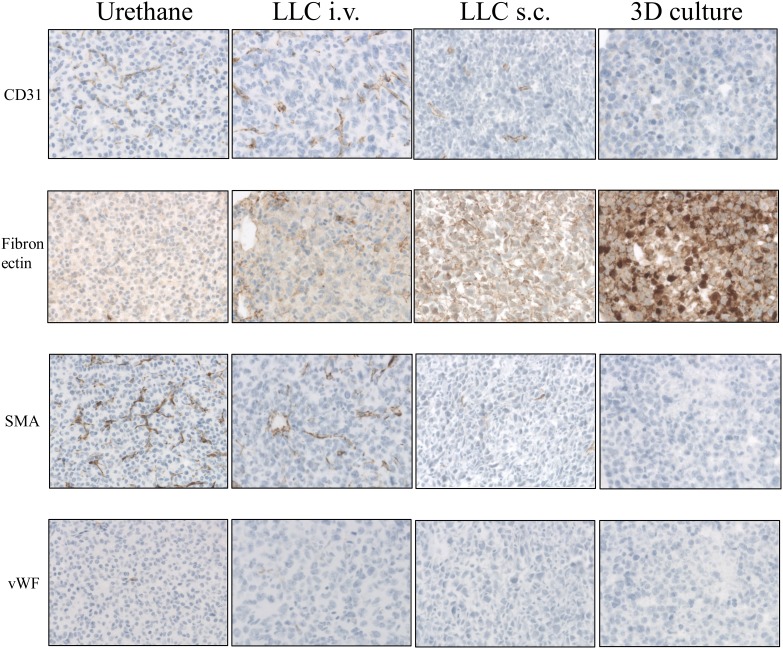
Immunohistological analysis of animal lung cancer models (x200 magnification for Urethane, LLC i.v., LLC s.c. and for 3D primary cell culture models): Urethane, LLC i.v., LLC s.c. induced lung tumor samples and 3D primary cell culture model spheroids were stained by antibodies of CD31, Fibronectin, SMA and vWF

All *in vivo* models of lung cancer consistently developed a comparable tumor with an incidence rate of 100%. The induction period of the Urethane-induced tumor was significantly longer than in other models (20 weeks v.s. 2 weeks respectively). However, TLOs were only found in the Urethane-induced tumor model. The immunohistological analysis (microscopy Figure 6A and 6B) revealed no significant count difference of infiltrated immune cells although CD4^+^ cells were found in higher numbers in the Urethane model. The 3D cell culture model showed fewer immune cells expressed in the spheroids after three days of culturing (Figure 6A and 6B). Tumor cells proliferated in all *in vivo* models consistently while tumor cells in spheroids of 3D culture model showed less proliferation. Table 1. provides a summary of the characteristics of the discussed models. We also compared the stromal composition of tumor models (Figure 7). An endothelial cell marker, CD31 was more expressed in Urethane and LLC i.v. model than s.c. or 3D culture model. The vWF, another endothelial cell protein, was weakly expressed in all models. Fibronectin, associated with cell adhesion and migration processes such as epithelial-mesenchymal transition, was expressed especially strong in the s.c. injection model and the 3D cell culture model, and less expressed in the urethane and i.v. injection models. Fibroblast and smooth muscle expressing smooth muscle actin (SMA) was found in both, the Urethane model and the LLC i.v. injection model.

**Table 1:** Comparison of model characteristics

Model	Incidence (%)	Induction period (weeks)	Morbidity (%)	Mortality (%)	CD4	CD8	F4/80	NKp46	TLO
**Urethane-injection model (orthotopic)**	100	20	0	0	++	++	+++	+	A
**Intravenous tumor cell line injection model (orthotopic)**	100	2	100 (Pleural effusion)	0 (2w) 60 (4w)	+	++	++	+	NA
**Subcutaneous tumor cell line injection model (heterotopic)**	100	2	0 (2w) 95 (3w)	0	+	++	+++	++	NA
**3D cell culture model (*ex vivo*)**	NA	NA	NA	NA	-	-	+	-	NA

Abbreviations: A: available; NA: not available; w: weeks; -: no positive cells; +: few present; ++: intermediately present; +++: abundantly present.

## DISCUSSION

We here compared three different preclinical lung cancer models, (I) the Urethane-induced primary lung cancer model, (II) the cell line-induced tumor model trough i.v. or s.c. injection and (III) the *ex vivo* 3D culture model of primary lung cancer. We compared these models with regard to tumor incidence, the duration of tumor induction and the composition of the immunohistological composition of the induced tumors. With the advent of the immune checkpoint inhibitors and the chimeric antigen receptor (CAR) T cells, cancer immunotherapies gain increased importance [14]. In the light of these emerging therapeutics, the characterization of relevant immune cells among most commonly used non-genetic mouse tumor models could be of help to optimize immunotherapy.

Urethane is used as a tumor-inducing agent in animal models, typically in inbred mice with a high susceptibility to spontaneous tumorigenesis. In contrast to the i.v. and s.c.-cell line injection models, the Urethane model takes more time for the development of tumors. However, this model provides a high tumor incidence rate and a low morbidity and mortality rate and was therefore considered a reliable tumor model. Moreover, up to 91% of the Urethane-induced mouse adenocarcinomas are bearing an activating mutations in the Kras oncogene which is a major advantage of the Urethane-induced tumor model [8]. Activating Kras mutations belong to the most frequent mutations in lung adenocarcinomas and can be found in about 17% of all lung adenocarcinoma patients. Up to now, there is no therapeutic intervention to target Kras mutation in non-small cell lung cancer (NSCLC). By using genomic profiling, Kras subgroups with varying therapeutic vulnerability have been identified and different therapeutic modalities are now tested on these subgroups [15]. Since the Kras mutation is an important property of the Urethane-induced tumor model, this model could be crucial in preclinical experiments.

TLO forms as a lymphatic tissue in the conditions of chronic inflammation, e.g. in autoimmune diseases, chronic infections or cancer and function as local sites for priming of the adaptive immune system (T and B cells) [16, 17]. The incidence of TLO is a prognostic marker for a better outcome in different cancer types [18–20]. Therefore, Urethane-induced tumor model comprises a high clinical relevance. Another carcinogen in-use is Diethylnitrosamine (DEN) which has the potential to induce tumors in various organs including the lung [21, 22]. We found in preliminary mouse experiments the tumor incidence rate to be significantly lower (3/10 mice) than in Urethane treated mice.

The s.c. cell line injection model was a reliable model with simple administration of tumor cells and conveniently accessible tumor nodules within the injection area. As the tumor nodules can easily be removed from beneath the skin, this model allows the sole analysis of the tumor tissue without the biasing adjacent lung or soft tissue. We found the morbidity rate highly depending on the area of injection, with a significant increased morbidity (skin ulcerations) in extended experiments when the cell line was injected into the neck area compared to injections into back area. This phenomenon was not demonstrated elsewhere. Therefore, if experiments are planned for a longer duration, cell line injections into neck area should be avoided.

In the i.v. injection model, orthotopic tumors were found homogenously distributed in the lung. Orthotopic tumor growth allows experiments on tumors in the clinically relevant site and reflects the physiologic setting in a more convenient way [23]. Since the surgical access to the inferior vena cava has to be carried out through the peritoneal cavity, this method is more challenging and insulting than the other models. In terms of tumor induction time and incidence rate, the i.v. injection model was comparable with the other s.c. injection models. However, in contrast to the other models, i.v. model showed a higher morbidity rate with complications such as pleural effusions or metastasis into the pleural cavity. However, this applies to the LLC cell line which we injected in this tumor model, other cell lines might have a different behavior with regard to side effects, but also to growth rate, induction period or tumor incidence rate.

We compared the immunohistological results of the *in vivo* mouse models with an *in vitro* model, the 3D primary cell culture model. As a reliable alternative to the *in vivo* models, the 3D primary cell culture model proofed to be a feasible model in preclinical research [24, 25] as well as in the characterization of an individual tumor [26–30]. With their more physiological tumor microenvironment, cell-matrix and cell-cell communications and gradients of oxygen, nutrients and growth factors, the 3D cell culture models feature a more sophisticated method for *ex vivo*/*in vitro* studies than the conventional 2D cell culture models [12, 29]. While T cells were consistently expressed in the other models, most prominent in the i.v. injection of LLC, the immunohistological examination of 3D cell culture model showed CD4^+^ and CD8^+^ cells to be almost absent in tumor spheroids built from cells of s.c.-LLC injection tumor.

With regard to the shortcomings of this study, we emphasize that we did not consider the patient derived xenograft model, reflecting the human gene expression status, the drug response and the tumor architecture of the patients’ tumor cells [31]. Another drawback is the fact that immune cells were virtually absent three days after culturing and the proliferation arrested after seven days of culturing in the spheroids of our 3D primary culture model.

In conclusion, the different discussed lung tumor mouse models showed a similar high tumor incidence rate after injection of tumor cells, but differed in terms of induction time, morbidity and mortality. Though there were tendencies in immunohistochemical staining of immune cells, no clear significant difference in the count of tumor infiltrating immune cells could be found. It can therefore be expected that when testing for immunotherapies in these models, results on them can be considered to be comparable. However, TLOs could exclusively found in the Urethane-induced tumor model which should be considered in experiments involving the adaptive immune system.

## MATERIALS AND METHODS

### Animals and animal care

Male wild type mice (BALB/c and C57BL/6, Charles River, Germany) were used for all experiments. Animals were fed a standard laboratory diet with water *ad libitum* and were kept under constant environmental conditions in the Biological Central Labor, University Hospital Zurich. All experimental procedures were approved by the veterinary office of the canton of Zurich (License number: ZH83/14) and performed in accordance with the institutional animal care guidelines.

### Tumor cell line

Lewis lung carcinoma (LLC) cell line was purchased from American Type Culture Collection (Manassas, USA). The cell line was stored at early passages (<3) in liquid nitrogen and were used in the experiments for no more than 6 months. The cell line was cultivated in DMEM containing 10 % FBS and penicillin/streptomycin within 5 % CO_2_ chamber.

### Urethane-induced tumor model

Two experimental groups of C57BL/6 mice were formed: one group received one intraperitoneal (i.p.) injection with 1 mg/g Urethane (Sigma, Germany); the other group received three i.p. injections with 1 mg/g Urethane in one week. Twenty weeks after injection, the animals were sacrificed by exsanguination, followed by flushing with saline and *en bloc* resection of the thoracic organs including bilateral lungs, heart, and thymus. The whole lung was fixed in formalin and embedded in paraffin for counting tumor nodules and immunohistochemistry (IHC).

### Cell line-induced tumor model via intravenous injection of LLC

LLC cells (0.25 x 10^6^ cells/mouse) were injected into the inferior vena cava of C57BL/6 mice after midline laparotomy under isoflurane anesthesia. Under the control of the vital signs (e.g. respiration rate, organ color) and integrity of the vessel at the injection site, the abdominal wall was closed by a running suture. Two weeks after tumor cell injection, total lungs were weighted. Animals were sacrificed by exsanguination followed by flushing with saline and *en bloc* resection of thoracic organs including bilateral lungs, heart, and thymus.

### Cell line induced tumor model via subcutaneous injection of LLC

LLC cells (1 x 10^6^ cells/mouse) were injected under the skin of C57BL/6 mice (either back or neck area) within serum free DMEM. Two weeks after tumor cell injection, the tumor was isolated from skin and weighed after exsanguination according to the experimental time points. The tumor mass was kept in formalin and embedded in paraffin for IHC.

### *Ex vivo* 3D primary tumor cell culture model

The primary tumor developed by subcutaneous (s.c.) injection of LLC cell line was harvested two weeks after inoculation and was digested by collagenase. Chopped tumor tissue was digested in buffer-containing collagenase II for one hour and passed through 30um pore size mesh. After washing, single cell suspension was placed with ACL-4 media. The cell suspension of the primary tumor was counted by a TC-20 automatic cell counter (Biorad) and placed onto 3D culture plates including a Perfecta hanging drop plate (Sigma, Germany) using 10^4^ cells. The media was changed daily. After five days, tumors were fixed in 4% paraformaldehyde and fixed with Histogel and paraffin for IHC.

### Histology and immunohistochemistry

Formalin fixed and paraffin embedded samples were stained by antibodies against Ki-67 (Abcam, UK), CD4 (eBioscience, USA), CD8 (eBioscience, USA), F4/80 (BMA Biomedicals, Switzerland), NKp46 (R&D Systems, USA), CD31 (Abcam, UK), fibronectin (Millipore, Switzerland), smooth muscle actin (SMA, Dako, USA), and von Willebrand Factor (vWF, Dako USA). The evaluation of stainings was conducted in a blind manner. For the microscopic assessment of Urethane-induced tumors, we counted tumor nodules in all lungs embedded in a horizontal direction on paraffin. The size of tumor nodules was measured at x40 magnification with a microscope (Leica, Wetzlar, Germany) and extrapolated into actual size. TLOs were counted in all lungs. TLOs are lymphatic tissues postnatally induced through inflammation or infection in non-lymphoid organs and functioning as sites for priming of the adaptive immune system (T and B cells) [16].

### Statistical analysis

Data were presented as means ± SD. Groups were compared with the Student t-test for unpaired samples using Prism 4.0 (GraphPad Software, San Diego, CA, USA). A two-sided p-value <0.05 was considered as statistically significant.

## References

[1] Cekanova M, Rathore K (2014). Animal models and therapeutic molecular targets of cancer: utility and limitations. Drug Des Devel Ther.

[2] Ruggeri BA, Camp F, Miknyoczki S (2014). Animal models of disease: pre-clinical animal models of cancer and their applications and utility in drug discovery. Biochem Pharmacol.

[3] Stiedl P, Grabner B, Zboray K, Bogner E, Casanova E (2015). Modeling cancer using genetically engineered mice. Methods Mol Biol.

[4] Sharma SV, Haber DA, Settleman J (2010). Cell line-based platforms to evaluate the therapeutic efficacy of candidate anticancer agents. Nat Rev Cancer.

[5] Vikis HG, Rymaszewski AL, Tichelaar JW (2013). Mouse models of chemically-induced lung carcinogenesis. Front Biosci (Elite Ed).

[6] Tuveson DA, Jacks T (1999). Modeling human lung cancer in mice: similarities and shortcomings. Oncogene.

[7] Gurley KE, Moser RD, Kemp CJ (2015). Induction of Lung Tumors in Mice with Urethane. Cold Spring Harb Protoc.

[8] You M, Candrian U, Maronpot RR, Stoner GD, Anderson MW (1989). Activation of the Ki-ras protooncogene in spontaneously occurring and chemically induced lung tumors of the strain A mouse. Proc Natl Acad Sci U S A.

[9] Cazorla M, Hernández L, Fernández PL, Fabra A, Peinado MA, Dasenbrock C, Tillmann T, Kamino K, Campo E, Kohler M, Morawieltz G, Cardesa A, Tomatis L, Mohr U (1998). Ki-ras gene mutations and absence of p53 gene mutations in spontaneous and urethane-induced early lung lesions in CBA/J mice. Mol Carcinog.

[10] Richmond A, Su Y (2008). Mouse xenograft models vs GEM models for human cancer therapeutics. Dis Model Mech.

[11] Lottner C, Knuechel R, Bernhardt G, Brunner H (2004). Distribution and subcellular localization of a water-soluble hematoporphyrin-platinum(II) complex in human bladder cancer cells. Cancer Lett.

[12] Edmondson R, Broglie JJ, Adcock AF, Yang L (2014). Three-dimensional cell culture systems and their applications in drug discovery and cell-based biosensors. Assay Drug Dev Technol.

[13] Wang C, Tang Z, Zhao Y, Yao R, Li L, Sun W (2014). Three-dimensional *in vitro* cancer models: a short review. Biofabrication.

[14] Yang Y (2015). Cancer immunotherapy: harnessing the immune system to battle cancer. J Clin Invest.

[15] Garrido P, Olmedo ME, Gómez A, Paz Ares L, López-Ríos F, Rosa-Rosa JM, Palacios J (2017). Treating KRAS-mutant NSCLC: latest evidence and clinical consequences. Ther Adv Med Oncol.

[16] Weinstein AM, Storkus WJ (2016). Biosynthesis and Functional Significance of Peripheral Node Addressin in Cancer-Associated TLO. Front Immunol.

[17] Neyt K, Perros F, GeurtsvanKessel CH, Hammad H, Lambrecht BN (2012). Tertiary lymphoid organs in infection and autoimmunity. Trends Immunol.

[18] Di Caro G, Bergomas F, Grizzi F, Doni A, Bianchi P, Malesci A, Laghi L, Allavena P, Mantovani A, Marchesi F (2014). Occurrence of tertiary lymphoid tissue is associated with T-cell infiltration and predicts better prognosis in early-stage colorectal cancers. Clin Cancer Res.

[19] Behr DS, Peitsch WK, Hametner C, Lasitschka F, Houben R, Schönhaar K, Michel J, Dollt C, Goebeler M, Marx A, Goerdt S, Schmieder A (2014). Prognostic value of immune cell infiltration, tertiary lymphoid structures and PD-L1 expression in Merkel cell carcinomas. Int J Clin Exp Pathol.

[20] Hiraoka N, Ino Y, Yamazaki-Itoh R, Kanai Y, Kosuge T, Shimada K (2015). Intratumoral tertiary lymphoid organ is a favourable prognosticator in patients with pancreatic cancer. Br J Cancer.

[21] Park DH, Shin JW, Park SK, Seo JN, Li L, Jang JJ, Lee MJ (2009). Diethylnitrosamine (DEN) induces irreversible hepatocellular carcinogenesis through overexpression of G1/S-phase regulatory proteins in rat. Toxicol Lett.

[22] Verna L, Whysner J, Williams GM (1996). N-nitrosodiethylamine mechanistic data and risk assessment: bioactivation, DNA-adduct formation, mutagenicity, and tumor initiation. Pharmacol Ther.

[23] Bibby MC (2004). Orthotopic models of cancer for preclinical drug evaluation: advantages and disadvantages. Eur J Cancer.

[24] Knight E, Przyborski S (2015). Advances in 3D cell culture technologies enabling tissue-like structures to be created *in vitro*. J Anat.

[25] Suggitt M, Bibby MC (2005). 50 years of preclinical anticancer drug screening: empirical to target-driven approaches. Clin Cancer Res.

[26] Gu L, Liao Z, McCue P, Trabulsi EJ, Nevalainen MT (2013). *Ex vivo* prostate cancer explant organ culture model system for targeted drug development in prostate cancer. J Clin Oncol.

[27] Meijer TG, Naipal KA, Jager A, van Gent DC (2017). *Ex vivo* tumor culture systems for functional drug testing and therapy response prediction. Future Sci OA.

[28] Antoni D, Burckel H, Josset E, Noel G (2015). Three-dimensional cell culture: a breakthrough *in vivo*. Int J Mol Sci.

[29] Weiswald LB, Bellet D, Dangles-Marie V (2015). Spherical cancer models in tumor biology. Neoplasia.

[30] Ivanov DP, Parker TL, Walker DA, Alexander C, Ashford MB, Gellert PR, Garnett MC (2015). *In vitro* co-culture model of medulloblastoma and human neural stem cells for drug delivery assessment. J Biotechnol.

[31] Reyal F, Guyader C, Decraene C, Lucchesi C, Auger N, Assayag F, De Plater L, Gentien D, Poupon MF, Cottu P, De Cremoux P, Gestraud P, Vincent-Salomon A (2012). Molecular profiling of patient-derived breast cancer xenografts. Breast Cancer Res.

